# Cranial variability and differentiation among golden jackals (*Canis aureus*) in Europe, Asia Minor and Africa

**DOI:** 10.3897/zookeys.917.39449

**Published:** 2020-03-09

**Authors:** Stoyan Stoyanov

**Affiliations:** 1 Wildlife Management Department, University of Forestry, Sofia, Bulgaria, 10 St. Kliment Ohridski Blvd., 1797, Sofia, Bulgaria University of Forestry Sofia Bulgaria

**Keywords:** Canid, *C. anthus*, *C. aureus*, *C. aureus moreotica*, *C. lupaster*, morphology, skull morphometry, taxonomy

## Abstract

Golden jackal (*Canis aureus*) expansion in the last decades has triggered research interest in Europe. However, jackal phylogeny and taxonomy are still controversial. Morphometric studies in Europe found differences between Dalmatian and the other European jackals. Recent genetic studies revealed that African and Eurasian golden jackals are distinct species. Moreover, large *Canis aureus lupaster* may be a cryptic subspecies of the African golden jackal. Although genetic studies suggest changes in *Canis aureus* taxonomy, morphological and morphometric studies are still needed. The present study proposes the first comprehensive analysis on a wide scale of golden jackal skull morphometry. Extensive morphometric data of jackal skulls from Europe (including a very large Bulgarian sample), Asia Minor, and North Africa were analysed, by applying recently developed statistical tools, to address the following questions: (i) is there geographic variation in skull size and shape among populations from Europe, Anatolia and the Caucasus?, (ii) is the jackal population from the Dalmatian coast different?, and (iii) is there a clear distinction between the Eurasian golden jackal (*Canis aureus*) and the African wolf (*Canis lupaster* sensu lato), and among populations of African wolves as well? Principal component analysis and linear discriminant analysis were applied on the standardized and log-transformed ratios of the original measurements to clearly separate specimens by shape and size. The results suggest that jackals from Europe, Anatolia and the Caucasus belong to one subspecies: *Canis aureus moreotica* (I. Geoffroy Saint-Hilaire, 1835), despite the differences in shape of Dalmatian specimens. The present study confirmed morphometrically that all jackals included so far in the taxon *Canis aureus* sensu lato may represent three taxa and supports the hypothesis that at least two different taxa (species?) of *Canis* occur in North Africa, indicating the need for further genetic, morphological, behavioural and ecological research to resolve the taxonomic uncertainty. The results are consistent with recent genetic and morphological studies and give further insights on golden jackal taxonomy. Understanding the species phylogeny and taxonomy is crucial for the conservation and management of the expanding golden jackal population in Europe.

## Introduction

The golden jackal (*Canis aureus* Linnaeus, 1758) is one of the most widely distributed canid species and is found in many areas of Europe, Asia and Africa (Jhala and Moehlman 2004; Arnold et al. 2012; Hoffmann et al. 2018; Moehlman and Hayssen 2018; Spassov and Acosta-Pankov 2019). Since the 1980s jackals have increased in their distribution and abundance in what is arguably the most dramatic recent expansion in Europe among native predators on the continent, and today the species is widespread throughout southern Asia, the Middle East and south-eastern and central Europe, where jackals inhabit a wide variety of habitats, from semi-deserts and grasslands to forested, agricultural, and semi-urban habitats (Jhala and Moehlman 2004; Šálek et al. 2014; Koepfli et al. 2015; Trouwborst et al. 2015). The jackal expansion in the last two decades was rapid and still ongoing. Jackals have expanded into Switzerland, Germany, Poland, Denmark, Netherlands and the Baltics (Pyšková et al. 2016; Potočnik et al. 2019). The ongoing expansion of the species in Europe has caused concerns regarding possible negative effects its presence could exert, due to excessive predation of other wildlife species or livestock, and the transmission of pathogens (Rutkowski et al. 2015; Ćirović et al. 2016). In addition, there are several uncertainties regarding jackal management and policies, often in association with the unknown origins of jackal populations (Trouwborst et al. 2015).

Jackal expansion in the last decades has triggered research interest in Europe. Many aspects of golden jackal’s ecology, diet, population density, genetics, legal implications of range expansion and management have been studied thoroughly in Europe. However, jackal phylogeny and taxonomy are still controversial. As many as 13 subspecies of golden jackal have been distinguished historically, but taxonomic revision is needed (Moehlman and Hayssen 2018). Recent genetic analyses revealed that *Canis aureus* from Africa should be considered as a separate species more closely allied to the wolf, *Canis lupus* Linnaeus, 1758 (Koepfli et al. 2015; Gopalakrishnan et al. 2018). Koepfli et al. (2015) suggested the name *Canis anthus* (Cuvier, 1820) for the African golden jackal. In addition, large *Canis aureus* in Egypt (*C. aureus lupaster* (Hemprich & Ehrenberg, 1833)) may be a cryptic subspecies of *Canis anthus* (Rueness et al. 2011; Gaubert et al. 2012). Traditionally, *C. aureus lupaster* is referred to as a golden jackal. However, Ferguson (1981) suggested that the taxon *C. aureus lupaster*, which is present in arid areas of Egypt and Libya, may represent a small *Canis lupus* rather than a large jackal. The opinion that *Canis lupaster* must be considered as a different species was recently confirmed by other studies based on morphological differences (Spassov and Stoyanov 2014; Bertè 2017; Viranta et al. 2017). However, recently published accounts on the issue (Rueness et al. 2011; Gaubert et al. 2012; Koepfli et al. 2015; Viranta et al. 2017; Gopalakrishnan et al. 2018) proved the need for morphological and morphometric studies to resolve taxonomic uncertainty. According to Moehlman and Hayssen (2018), all jackals included so far in the taxon *Canis aureus* may represent three canid taxa: *Canis aureus*, *Canis anthus* and *Canis lupus*. However, based on recent genetic studies (Koepfli et al. 2015; Viranta et al. 2017; Gopalakrishnan et al. 2018), only *Canis lupaster* and *Canis aureus* are considered as valid taxa and *Canis lupaster* supersedes *Canis anthus* as a valid taxonomic name, although not widely accepted. Here I use the names “African golden jackal” (*Canis anthus* s. str.) and “African wolf” (*Canis lupaster* s. str.) for identification purposes, in order to separate samples of the larger wolf-like canid skulls from other medium-sized skulls of African canid species (see also Kryštufek and Tvrtković 1990; Bertè 2017).

Craniometric differentiation of golden jackal in Europe has been so far poorly studied. While genetic studies are increasing, recent papers on cranial morphometry are still scarce and describe local populations. Morphometric analyses of museum specimens have shown that jackals from Dalmatia appeared to be morphologically well distinct from their counterparts from the Balkan Peninsula and Africa, with the greatest similarity to the jackals from Asia Minor (Kryštufek and Tvrtković 1990). Recent studies on craniometrical relationship patterns of jackal populations from Hungary, Bulgaria, and Serbia displayed no significant differences between the Balkan Peninsula and Pannonia, except in some age groups (Markov et al. 2017; Krendl et al. 2018). Geometric morphometric analyses in Croatia confirmed slight morphological variation in jackal skulls (Rezić et al. 2017). Genetic studies focused on jackals in Bulgaria, Serbia, Croatia and Italy suggested a low level of genetic diversity and weakly pronounced genetic structure, with only the coastal population from Dalmatia clearly differentiated from other Balkan samples (Zachos et al. 2009; Fabbri et al. 2014).

Morphometric relationships of the European golden jackals with jackals from the Asiatic part of the species’ range have not yet been determined. Moreover, none of the studies so far have analysed morphometrically jackal populations on a larger scale. Consequently, the understanding of historic development of jackal populations in Europe is lacking (Rutkowski et al. 2015). The claim that jackals were already present along the Mediterranean coast in Croatia and Greece ca 7000–6500 years BP (Sommer and Benecke 2005), although widely cited (e.g., Zachos et al. 2009; Rutkowski et al. 2015; Trouwborst et al. 2015, Krofel et al. 2017; Lanszki et al. 2018) is more than doubtful, as it is based on remains whose taxonomic affinities are uncertain (Spassov and Acosta-Pankov 2019). The most comprehensive continent-wide genetic study in Europe so far (Rutkowski et al. 2015) supports the hypothesis that an ancient Greek population survived in the Peloponnese to the present day, recently merging with a population expanding in from the east, and a similar interpretation can be put forward in regard to Dalmatian jackals, as suggested by Fabbri et al. (2014). Genetic analyses revealed that the Dalmatian coast and the Peloponnese are the only two areas in south-eastern Europe today that show higher genetic differentiation, giving further support for the continuous presence of ancient populations along the Mediterranean coast, and that there is ongoing gene flow between the Caucasus and Europe as well (Rutkowski et al. 2015). This hypothesis agrees with the opinion about jackal penetration into Eastern Europe from Anatolia or from the Caucasus in two ways that correspond to the potential paths at the end of Pleistocene and Holocene: along the northern Black Sea coast and through the Bosporus (Spassov 1989). According to Spassov (1989), the distribution area of the European subspecies *Canis aureus moreotica* (I. Geoffroy Saint-Hilaire, 1835) during the first half of 20^th^ century occupied a relatively vast territory from the Balkans, up to Anatolia and the Caucasus.

Bulgarian territory is considered the core area of golden jackal distribution in Europe with the highest population density (Stoyanov 2013; Spassov and Acosta-Pankov 2019). However, very few genetic studies include Bulgarian samples (e.g., Zachos et al. 2009; Fabbri et al. 2014; Yumnam et al. 2015). Morphometric studies, including skulls from Bulgaria, were very scarce and local so far (e.g., Markov et al. 2017; Krendl et al. 2018). The present study proposes the first comprehensive analysis on a wide scale of golden jackal skull morphometry. I analysed extensive morphometric data of jackal skulls from Europe, including a very large Bulgarian sample, Asia Minor and North Africa, by applying recently developed statistical tools to address the following questions: (i) is there geographic variation in skull size and shape among populations from Europe, Anatolia and the Caucasus?, (ii) is the jackal population from the Dalmatian coast different?, and (iii) is there a clear distinction between Eurasian golden jackal (*Canis aureus*) and African wolf (*Canis lupaster* sensu lato), and among populations of African wolves as well? Although genetic studies suggest changes in *Canis aureus* taxonomy, morphological and morphometric studies are still needed. Integration of genetic techniques and morphometrics represent a valuable tool in the resolution of taxonomic uncertainty. Here a craniometric perspective is offered.

## Material and methods

**Figure 1.**
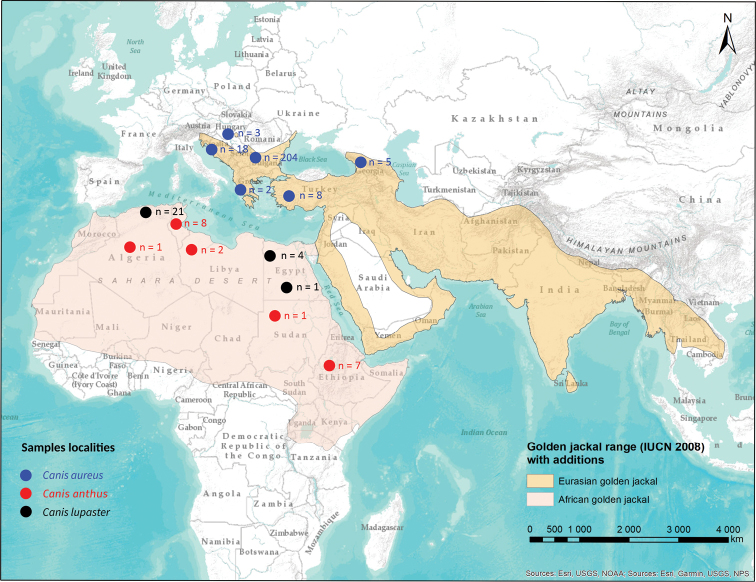
Map of Eurasian golden jackal range (Hoffmann et al. 2018) and African wolf range (Hoffmann and Atickem 2019) based on IUCN Red List data. Sample localities (countries) and number of measured specimens are shown. Note: The range map of Eurasian golden jackal from IUCN Red List has not been updated since 2008. The confirmed presence of jackals in Poland, Denmark, Netherlands and Baltic countries is not shown.

I morphometrically compared a total of 285 skulls of Eurasian golden jackal (*Canis aureus*) from Europe and Asia Minor and African wolf (*Canis lupaster* sensu lato) from North Africa. Most of the jackal skulls were collected in Bulgaria. This sample included 198 jackal skulls from subadult and adult golden jackals. Juvenile specimens were defined as individuals with fully developed second dentition, but less than 10 months of age; subadults as individuals more than 10 months, when they reach sexual maturity, but less than two years of age; and adults as two years and older. I determined the age in consideration of upper incisive teeth wear (Lombaard 1971) and for some individuals also by counting the annual cementum layers in canines (Klevezal and Kleinenberg 1967). Both methods are reliable enough for the purposes of the study and provide accurate results, with precision up to one year for the first one (Harris et al. 1992, Raichev 2002). Although there are some differences in size between juveniles, subadults and adult jackals, e.g. in condylobasal length, zygomatic breadth, mastoid breadth, the skulls of subadults and adult jackals could be hardly separated by shape (Stoyanov 2013). I used for comparisons also museum specimens and data published by other authors (Kryštufek and Tvrtković 1990; Demeter and Spassov 1993). Some museum specimens of subadult animals were included in the data analyses as well. The compared skulls were assigned to three different groups: *Canis aureus* (240 specimens) coming from Europe (Bulgaria, Greece, Hungary and Croatia) and Asia Minor (Turkey and the Caucasus), *Canis anthus* s. str. (19 specimens) from North Africa (Algeria, Tunisia, Libya, Sudan and Ethiopia), and *Canis lupaster* s. str. (26 specimens) from Algeria, Sudan and Egypt (Fig. 1).

### Morphometric comparison

**Figure 2.**
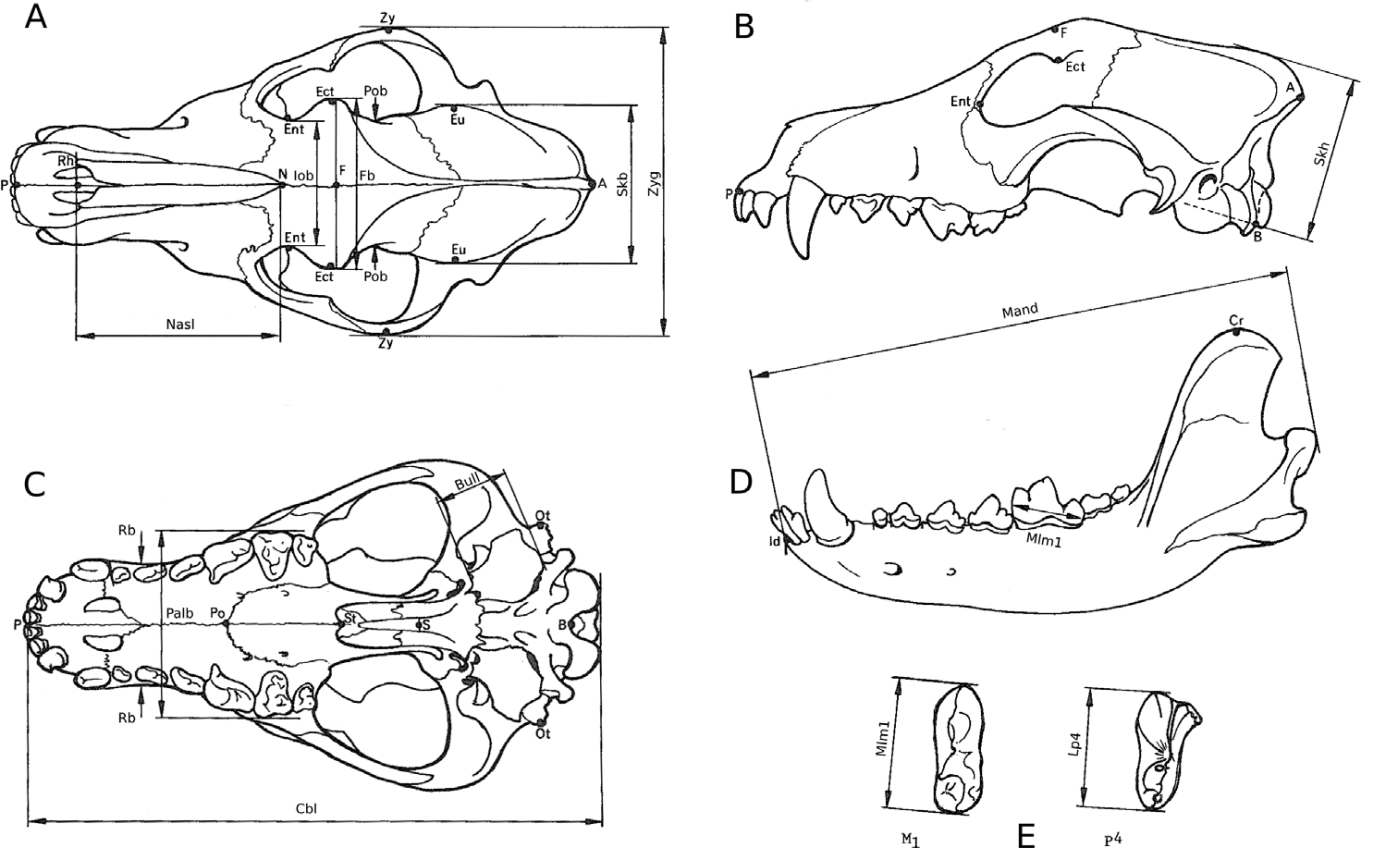
Skull measurements employed in the analyses (following von den Driesch 1976): condylobasal length (Cbl), greatest length of the nasals (Nasl), length of the carnassial (P^4^), measured at the cingulum (Lp4), greatest diameter of the auditory bulla (Bull), greatest breadth of the braincase (Skb), zygomatic breadth (Zyg), least breadth at the postorbital constriction (Pob), frontal breadth (Fb), least breadth between the orbits (Iob), greatest palatal breadth (Palb), least palatal breadth (Rb), skull height (Skh), total length of the mandible (Mand) and length of the carnassial (M_1_), measured at the cingulum (Mlm1). **A***Canis* cranium, dorsal view **B***Canis* cranium, left side view **C***Canis* cranium, basal view **D***Canis* mandible, left side, lateral view **E***Canis* upper and lower carnassial (P^4^ and M_1_).

Fourteen skull measurements, following von den Driesch (1976), twelve cranial and two of the mandibles, from 285 skulls were taken (Fig. 2). I focused only on the most widely accepted and frequently measured craniodental measurements that have been used by previous authors and could be compared among different publications: condylobasal length (Cbl), greatest length of the nasals (Nasl), length of the carnassial (P^4^), measured at the cingulum (Lp4), greatest diameter of the auditory bulla (Bull), following Wagner (1930), greatest breadth of the braincase (Skb), zygomatic breadth (Zyg), least breadth at the postorbital constriction (Pob), according to Duerst (1926), frontal breadth (Fb), least breadth between the orbits (Iob), greatest palatal breadth (Palb), least palatal breadth (Rb), skull height (Skh), following (Wagner 1930), total length of the mandible (Mand) and length of the carnassial (M_1_), measured at the cingulum (Mlm1). I measured personally by using a digital sliding calliper 221 skulls (198 specimens of *Canis aureus* from Bulgaria, two specimens of *Canis aureus* from the Caucasus, and 21 specimens of *Canis lupaster* s. str. from Algeria). Although the precision of the calliper was 0.01 mm, all craniodental measurements were taken with precision up to 0.1 mm. The measurements of the other 64 skulls used in the analyses (40 specimens of *Canis aureus* from Europe, Anatolia and the Caucasus, 19 specimens of *Canis anthus* s. str. and five specimens of *Canis lupaster* s. str. from North Africa) were published by other authors (Kryštufek and Tvrtković 1990; Demeter and Spassov 1993, see Suppl. material 1).

### Statistical methods

All measurements were tested for normality by QQ plots and the Shapiro-Wilk test. I applied multivariate analyses in order to explore the most significant variation in size and shape of the skulls. Shape in general tends to provide more reliable information than size on the morphology of organisms (Jolicoeur and Mosimann 1960). Size is often considered as a nuisance because it is strongly dependent on ecological factors (McCoy et al. 2006), but separation of size and shape in multivariate studies of morphological data is problematic (Claude 2008). I addressed this problem by using principal component analysis (PCA). The first principal component of PCA is usually considered as a general size axis, while the remaining principal components represent the shape space. However, it also includes size-related shape information (Jolicoeur and Mosimann 1960) and has been identified by Jolicoeur (1963) heuristically as a multivariate allometric size axis. The mixture of size and size-related shape information in the first component makes the interpretation of the other components of a PCA rather difficult. New methods have been developed recently allowing interpretation of principal components in terms of ratios and clear separation of size and shape (Baur and Leuenberger 2011). These authors defined an isometric size axis (called “isosize”, see Baur and Leuenberger 2011) as the geometric mean of the original measurements and thus comprising only differences in scaling. We could obtain allometry-free shape variables by projecting the measurements orthogonal to isosize. A PCA calculated on the covariance matrix of these shape variables then accounts solely for differences in proportions. Baur and Leuenberger (2011) suggested to plot the isosize against each significant shape component in order to assess the amount of allometry in the data.

The advantages of ratios are that their computation is simple, and that one can easily interpret them in geometric terms of shape variation (Claude 2008). However, several authors have pointed out that working with ratios introduces spurious correlations between variables (Atchley et al. 1976; Atchley and Anderson 1978; Claude 2008), data becomes dependent after being standardized leading to the increase of correlation, and scaling affects the geometry of the shape space, so that it becomes non-Euclidean (Claude 2008). Although it removes the size parameter, using ratios increases the correlation between data. Ratios may pose difficult problems for multivariate statistical methods because of the curious distributions that they sometimes possess, but given that such problems can be overcome, they may be one of the best ways to deal with simple size, which may explain why studies using ratios have been so successful (Oxnard 1978). A second way to conceptualize shape and size is to consider shape as the remaining variation once variation explained by size has been filtered. Shape will correspond to the residual variance. This approach has the disadvantage of being more difficult than the former one for understanding variation in geometric terms (Claude 2008). In contrast to linear measurements, the geometric morphometric approach provides unbiased descriptions of shape as well as helping to quantify selection on different craniodental traits, but this method still has some problems, e.g., choosing the right landmarks and difficulties in analysing three-dimensional shapes (see Claude 2008). However, it was not possible to employ it in the present study, because not all skulls were available for measurement.

For clear separation of shape and size, the PCA was applied on the standardized (dividing each measurement by geometric mean) and log-transformed ratios of the original measurements (Claude 2008; Baur and Leuenberger 2011). To examine how well the skulls of different taxonomic groups are separated, the data were subjected to a linear discriminant analysis (LDA). The performance of the LDA was assessed by means of cross validation (Rencher 2002), where one specimen is omitted from the analysis and classified according to the discriminant function found for the remaining specimens in the data set.

Geometric interpretation of PCA and LDA was made by using graphical tools developed by Baur and Leuenberger (2011). I applied the “PCA ratio spectrum” for the interpretation of principal components in shape space, and the “LDA ratio extractor” for finding the best ratios that separate the skulls of different taxonomic groups. The amount of allometry in the data was assessed by the “allometry ratio spectrum”.

For detailed mathematical descriptions and statistical frameworks of the applied methods see Claude (2008) and Baur and Leuenberger (2011). All statistical and graphical analyses were performed with R, version 3.6.1 (R Core Team 2019). Slightly modified versions of the R-scripts provided by Baur and Leuenberger (2011) and Claude (2008) were employed for calculations. PCA and LDA were performed using the MASS software package (Venables and Ripley 2002).

### Ethics statement

The skull samples used in this study were obtained from individuals that died in vehicle collisions, due to natural causes or as a result of legal hunting. I also measured museum specimens. No animal was killed for the purpose of this study.

## Results

**Figure 3.**
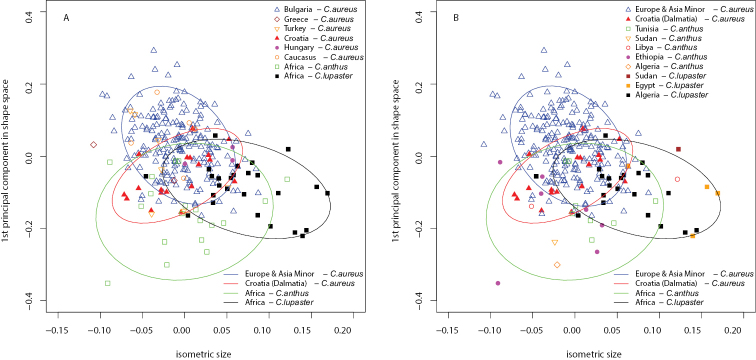
Principal component analysis. Separation between taxonomic groups along isometric size and first principal components in shape space. Ellipses show 95 % confidence interval for each group. Specimens of *Canis aureus* are divided in two groups – Europe and Asia Minor, and Croatia (the Dalmatian coast). **A** country origin of European specimens is marked with different symbols **B** country origin of African specimens is presented.

Shapiro-Wilk tests and QQ plots showed that all measurements did not deviate significantly from a normal distribution. However, for most of the following statistical methods the assumption of normally distributed data is not strongly suggested. PCA revealed that there was a clear separation between the predefined taxonomic groups. Four clusters could be differentiated projecting the data along isosize and the first principal component in shape space: European golden jackals, including Anatolia and the Caucasus, Dalmatian jackals, and two groups of African wolves (in the broad sense) (Fig. 3). Most of the skulls of *Canis aureus* are from Bulgaria, but there is no clear separation between the Bulgarian jackals and the specimens from Greece, Hungary, Turkey, and the Caucasus (Fig. 3A). The European jackals form the most homogeneous cluster on the plot as it is shown by the ellipses enclosing 95% of the confidence interval for each taxonomic group. Only the Dalmatian jackals show differences in shape along the first principal component, but not in size. Both groups of African wolves (in the broad sense) were clearly distinguished as well. The African jackals (*Canis anthus* s. str.) form homogenous cluster despite the different origins of the specimens (Fig. 3B). There are no differences in shape and size of skull among jackals from Libya, Tunisia, Sudan and Ethiopia. However, the African specimens could be easily separated from the Eurasian specimens by their skull shape. The skulls of *Canis lupaster* s. str. are bigger than the skulls of *Canis aureus* and *Canis anthus* s. str. and could be easily separated by shape as well.

**Figure 4.**
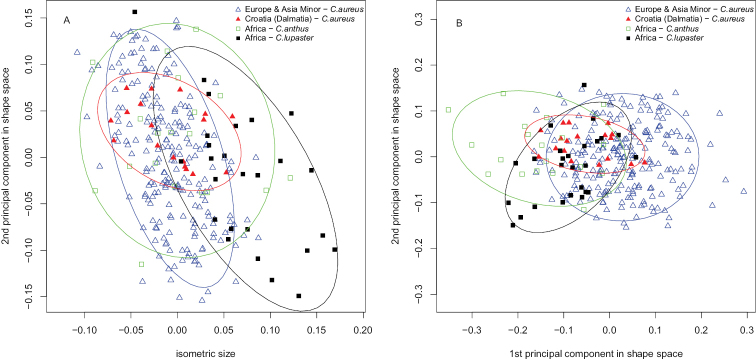
Principal component analysis. The first two principal components in shape space account for 53.6 % of the variance. Ellipses show 95 % confidence interval for each group. Specimens of *Canis aureus* are divided in two groups – Europe and Asia Minor, and Croatia (the Dalmatian coast). **A** separation between taxonomic groups along isometric size and second principal component **B** separation between taxonomic groups along first two principal components in shape space.

There are no clear differences in skull shape between the taxonomic groups revealed by the second shape principal component plotted against isosize (Fig. 4A). Only the skulls of *Canis lupaster* s. str. could be separated by their bigger size. The first two principal components in shape space accounted for 53.6 % of the variance (Fig. 4B). The four groups could be distinguished only along the first principal component, but with a large overlap in skull shape between clusters. Presence of allometry could be assessed while projecting the first shape principal component orthogonal to the isometric size (Fig. 3A). Judging from the graph, there is only a very moderate correlation between shape and size. Hence, allometric variation was of marginal importance for our data set.

**Figure 5.**
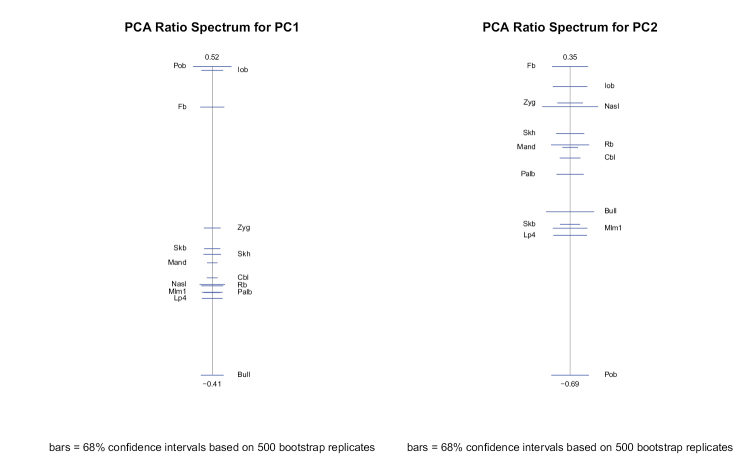
PCA ratio spectrum for the first and second principal component in shape space of the 14 craniodental measurements. See Material and methods and Figure 2 caption for the definition of the craniodental measurements.

**Figure 6.**
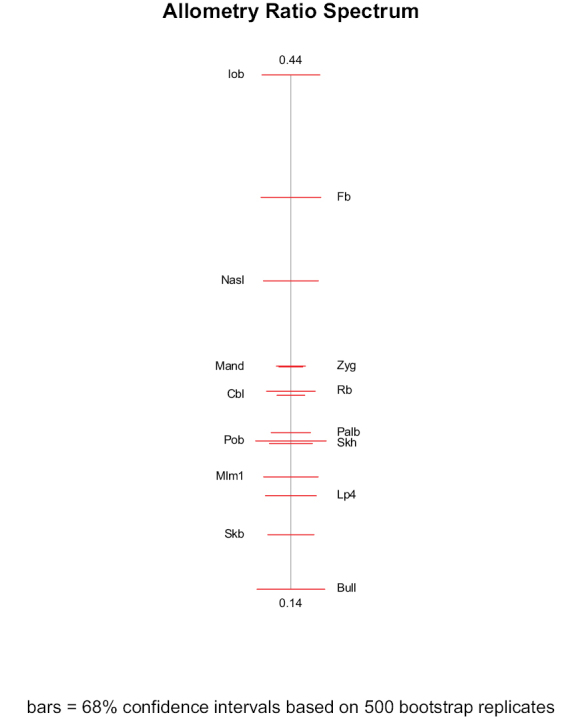
Allometry ratio spectrum of the 14 craniodental measurements used in this study. See Material and methods and Figure 2 caption for the definition of the craniodental measurements.

The “PCA ratio spectrum” allows the interpretation of principal components in shape space (Fig. 5). Considering factor loadings, ratios between least breadth at the postorbital constriction (Pob), least breadth between the orbits (Iob), and greatest diameter of the auditory bulla (Bull) explained a large proportion of the variance of the first shape principal component. The same ratios, however, showed the most distinctive allometric behaviour as could be seen from the “allometry ratio spectrum” (Fig. 6). Ratios between frontal breadth (Fb), least breadth between the orbits (Iob), and zygomatic breadth (Zyg), on the one side, and least breadth at the postorbital constriction (Pob), on the other side, contribute most to the variance of the second shape principal component. The PCA ratio spectrum is statistically stable because of the narrow confidence intervals shown on the graph.

**Figure 7.**
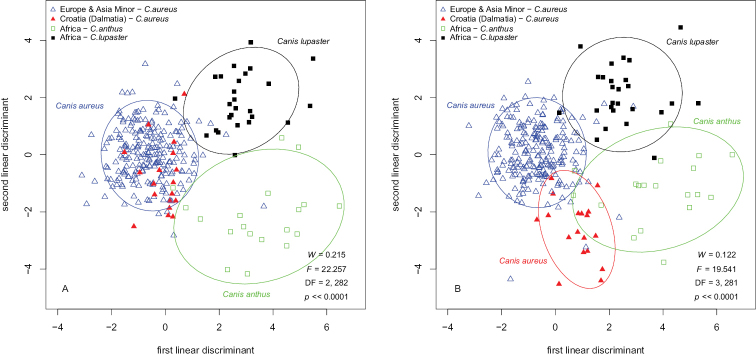
Separation of groups by LDA**A** three taxonomic groups were included in the analysis: Eurasian golden jackals, African golden jackals and African wolves **B** specimens from Dalmatia were included in the analysis as a separate group.

The results from PCA suggested that we could find the best separation of groups by employing LDA. The analyses were applied twice. First, I tried to discriminate the three taxonomic groups: Eurasian golden jackals, African golden jackals and African wolves. Next, I conducted analyses by including specimens from Dalmatia as a separate group, following the results from PCA and assumptions about the differences between Dalmatian jackals and their counterparts from the Balkan Peninsula and Africa, found by morphological and genetic studies so far. The LDA showed that skulls of *Canis aureus*, *Canis anthus* s. str. and *Canis lupaster* s. str. could be clearly distinguished (Fig. 7A). The performance of LDA was assessed by means of cross validation. Almost all skulls were correctly classified with very few exceptions (Table 1). The Mahalanobis distances between group centroids are almost identical, but the cluster of African wolves was closer to the cluster of Eurasian jackals (Table 2), and therefore more specimens between these two groups were misclassified. By applying LDA, although with inferior performance, I was able to separate clearly Dalmatian jackals as a distinct group (Fig. 7B). Again, most of the skulls were correctly classified (Table 3). As could be expected, the cluster of Dalmatian jackals was closer to the cluster of the other jackals from Europe and Asia Minor than to the African species (Table 4).

**Figure 8.**
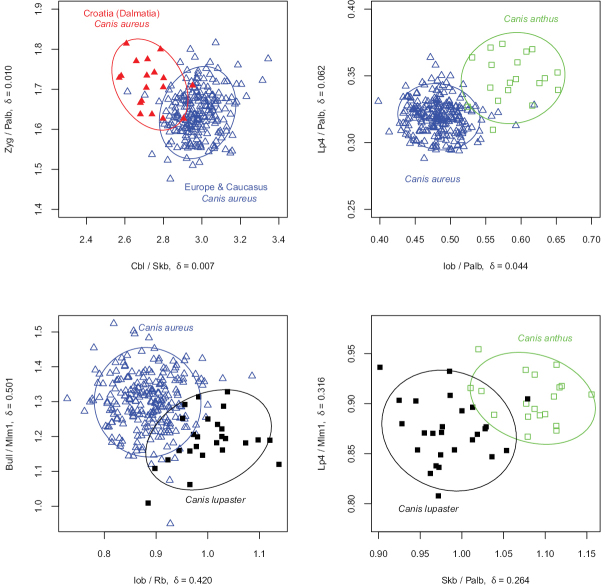
The ratios that best separated taxon groups revealed by the LDA ratio extractor. The measure *δ* indicates how well shape discriminates in relation to size. A value of *δ* close to unity means that separation is mainly due to size, whereas a value close to zero indicates separation is mainly due to shape.

For practical reasons, characters that would allow quick and easy identification of most specimens might be useful, for instance in field work. One or two ratios would be preferable, as these are easily calculated and differences in proportions can sometimes even be estimated by eye (Reichenbach et. al. 2012). Hence, I applied the LDA ratio extractor (Baur and Leuenberger 2011) to find the best ratios that could easily separate the skulls of *Canis aureus*, *Canis anthus* s. str., and *Canis lupaster* s. str. (Fig. 8).

The skulls of Dalmatian jackals are relatively broader overall, with a broader braincase, larger palatal and zygomatic breadth, and a shorter condylobasal length, compared to the skulls of jackals from Europe and Asia Minor. The differences are mostly in shape, but not in size of skulls. The ratio Iob/Palb very well separates the Eurasian from the African jackals, the latter also having a slightly longer upper carnassial (P^4^). These two groups are almost identical in size but with a different skull shape. *Canis lupaster* s. str. is well separated by *Canis aureus*, having a bigger Iob/Rb ratio and smaller diameter of the auditory bulla (Bull), in comparison to the length of the upper carnassial (P^4^). The skulls of *Canis lupaster* s. str. are bigger and broader, with a more elongated shape. The differences are both in size and shape of skulls. The ratios Skb/Palb and Lp4/Mlm1 best separate the *Canis lupaster* group from the *Canis anthus* group.

**Table 1.** Assessment of the LDA performance by cross validation. Number of specimens classified in each group.

Groups	Classified as:		
Original	*Canis aureus*	*Canis anthus* s. str.	*Canis lupaster* s. str.
*Canis aureus*	236	1	3
*Canis anthus* s. str.	3	14	2
*Canis lupaster* s. str.	5	0	21

**Table 2.** Results from the LDA. Distances between the group centroids.

Groups	*Canis aureus*	*Canis anthus* s. str.
*Canis anthus* s. str.	4.583	–
*Canis lupaster* s. str.	3.901	4.104

**Table 3.** Assessment of the LDA performance by cross validation. Number of specimens classified in each group.

Groups	Classified as:			
Original	*Canis aureus* (Europe & Asia Minor)	*Canis aureus* (Croatia – Dalmatia)	*Canis anthus* s. str.	*Canis lupaster* s. str.
*Canis aureus* (Europe & Asia Minor)	216	3	1	2
*Canis aureus* (Croatia – Dalmatia)	5	13	0	0
*Canis anthus* s. str.	3	0	14	2
*Canis lupaster* s. str.	5	0	0	21

**Table 4.** Results from the LDA. Distances between the group centroids.

Groups	*Canis aureus* (Europe & Asia Minor)	*Canis aureus* (Croatia – Dalmatia)	*Canis anthus* s. str.
*Canis aureus* (Croatia – Dalmatia)	3.574	–	–
*Canis anthus* s. str.	4.852	4.575	–
*Canis lupaster* s. str.	3.907	5.074	4.270

## Discussion

The results suggest that there is no clear differentiation among Eurasian jackals in skull size and shape. Although the sample size of Bulgarian jackals included in the analysis is the largest analysed to date, they form a homogenous cluster, but with large individual variability. Furthermore, there were hardly any differences in skull shape between the Bulgarian jackals and the specimens from Turkey, Greece, Hungary and the Caucasus. The Bulgarian jackal skulls encompass all other specimens from Hungary, Greece, Turkey and the Caucasus on the plots, as was revealed by PCA and LDA. The amount of geographical variation among the Eurasian jackals is comparable with sex and age differences within the entire Bulgarian subpopulation. However, the golden jackal skulls from Bulgaria showed also weak differentiation in size and shape, depending on the age and sex of the animals, despite their considerable individual variability (Stoyanov 2012). The Eurasian jackals form the most homogeneous cluster on the plots as was shown by the ellipses enclosing 95% of the confidence interval for each taxonomic group. The similarities in skull morphology and morphometrics between the jackals from Bulgaria, Serbia, Hungary and Austria were confirmed also by other studies (Markov et al. 2017; Krendl et al. 2018).

Only the Dalmatian jackals showed differences in shape, but not in size. Their skulls were easily separated by linear discriminant analyses and appeared to be broader and with shorter condylobasal length. Such differences were found also by other morphometric studies (Kryštufek and Tvrtković 1990; Stoyanov 2012) and are consistent with recent evidence, showing high level of genetic diversity and higher genetic differentiation of Dalmatian jackals (Zachos et al. 2009; Fabbri et al. 2014), and giving further support for the continuous presence of ancient populations along the Dalmatian coast (Fabbri et al. 2014; Rutkowski et al. 2015). These results could be due to a number of factors including historic changes in distribution, geographic isolation, founder effect for the isolated Dalmatian population, different ecological conditions, competition with grey wolves, and human pressure on golden jackal populations (Kryštufek and Tvrtković 1990; Zachos et al. 2009; Krofel et al. 2017; Newsome et al. 2017). Although the Dalmatian population is more distant morphologically and genetically from the other European populations (Kryštufek and Tvrtković 1990; Fabbri et al. 2014; Rutkowski et al. 2015), the results of the present study confirmed that the jackals from Dalmatia are closer morphometrically to their Eurasian counterparts than to the African jackals. However, it was possible to separate the Dalmatian jackals as a distinct group by tools of discriminant analysis.

The sample included only two museum specimens from Greece with their measurements published by Demeter and Spassov (1993). These specimens did not differ from the European cluster, but I did not have samples from the Peloponnesus Peninsula (southern Greece), where the existence of a genetically distinct population (Rutkowski et al. 2015) supports the hypothesis that an ancient Greek population survived in the Peloponnese to the present day, recently merging with a population expanding in from the east. However, this opinion is controversial (Spassov and Acosta-Pankov 2019).

All specimens from the Caucasus and Anatolia also fall into the cluster of Eurasian golden jackals and did not differ from Bulgarian, Hungarian and Greek skulls. These results were expected and confirm the hypothesis about jackal penetration in Eastern Europe from Anatolia or from the Caucasus in two ways, that correspond to the potential paths at the end of Pleistocene and Holocene: along the northern Black Sea coast and through the Bosporus (Spassov 1989). Recent genetic studies found that the Caucasus region harbours high genetic diversity in terms of the number of microsatellite alleles and there is ongoing gene flow between the Caucasus and Europe as well (Rutkowski et al. 2015). Moreover, the Caucasus region is known as a “hotspot” for biodiversity (Myers et al. 2000) and requires priority in the development of a conservation strategy for the golden jackal in Europe (Rutkowski et al. 2015). Furthermore, the current expansion to the continent has started from only three basal population nuclei: two from the Balkans (the Peri-Strandja area and the Dalmatian coast) and the Caucasus (Spassov and Acosta-Pankov 2019), which explains the morphometric similarities among Eurasian jackals.

Still, the question about differences between the Dalmatian jackals and the European population remains. It is clear, however, that there is no reason to consider these morphological differences as evidence for the existence of more than one subspecies on the Balkans and adjacent European countries. The subspecies *Canis aureus ecsedensis* (Kretzoi, 1947), or its synonym *Canis aureus hungaricus* Ehik, 1938 (Moehlman and Hayssen 2018), could not be justified as a separate subspecies in Europe, based on the present morphometric results. Genetic studies so far revealed that jackals in Europe are genetically similar, despite high level of genetic diversity and higher genetic differentiation in some European populations (Zachos et al. 2009; Fabbri et al. 2014; Rutkowski et al. 2015). The present morphometric study is consistent with the results of all recent genetic research in Europe and confirms the proposition that the jackals in Europe and the Caucasus belong to one subspecies *Canis aureus moreotica* (I. Geoffroy Saint-Hilaire, 1835), occupying a relatively vast territory from the Balkans, up to Anatolia and the Caucasus (Spassov 1989; Demeter and Spassov 1993). Moreover, there is no significant difference in the colouration pattern and other features across the various subpopulations living in this area (Pocock 1938; Heptner et al. 1967; Demeter and Spassov 1993). Although the subspecies *Canis aureus caucasica* Kolenati, 1858 was proposed as synonym of *Canis aureus aureus* Linnaeus, 1758 (Moehlman and Hayssen 2018), there is no evidence that the Caucasian jackals belong to this subspecies. Morphometric similarities found in the present study and the ongoing genetic flow between the Caucasus and Europe (Rutkowski et al. 2015) raises the question of the geographic border between *Canis aureus moreotica* and *Canis aureus aureus*. Furthermore, in previous studies I compared craniometrically Bulgarian jackals and their conspecifics inhabiting Amu Darya river lowlands in Uzbekistan using data published by other authors (Reimov and Nuratdinov 1970; Taryannikov 1974). Although I applied only univariate statistics, there were no significant differences in the main skull measurements between the jackals from Bulgaria and Middle Asia (Stoyanov 2013). However, morphometric studies alone cannot provide a basis for resolving the taxonomy and phylogenetic relationships, without the addition of genetic data. Three species of sympatric African jackals (*Canis lupaster*, *Lupulella adusta*, and *Lupulella mesomelas*), for example, are morphologically similar despite having diverged more than two million years ago, which could be explained by the greater diversity of predator and prey species in east Africa (Wayne et al. 1989).

Both PCA and LDA revealed clear differences between Eurasian golden jackals and the two groups of African wolves (*Canis lupaster* sensu lato). The results from PCA and LDA suggested the existence of significant morphological variation within *Canis lupaster* (in the broad sense). The population of African wolves was separated in two very distinct clusters both in size and shape of skulls. Although, there are significant differences in size between populations of *Canis lupaster*, with East African individuals being smaller than North and West African ones (Viranta 2017), it seems that this is not a clinal variation, and at least two different morphotypes exist (Gaubert et al. 2012, Saleh and Basuony 2014). A basicranial length distribution from 57 specimens identified in museums as *Canis aureus* and collected in North Africa, from Egypt to Morocco, is noticeably bimodal, with an anti-mode at around 160 mm, and a disproportionate number of the skulls (*N* = 35) measuring over 161.00 mm had the Nile Valley, and neighbouring areas, as their region of origin (and most of them, interestingly, were museumlabelled as *Canis aureus lupaster*) (Gonzalez 2012). The skulls of the *Canis lupaster* group in my study are bigger than the skulls of both *Canis aureus* and *Canis anthus* groups and could be easily separated by shape as well. The individuals of *Canis lupaster* group have broader skull with more elongated shape than the Eurasian and other African jackals. The differences are both in size and shape of skulls, and in some dental measurements as well. It does appear that both larger and smaller forms of *Canis lupaster* sensu lato, formerly known as subspecies of *Canis aureus* in Africa, occur sympatrically not only in Egypt and Libya, but also in Algeria and other North-African countries and they differ not only in their appearance, but in their behaviour and ecology as well (Gaubert et al. 2012, Saleh and Basuony 2014; Bertè 2017). Many authors consider *Canis lupaster* s. str. as a separate species (Kurtén 1974; Ferguson 1981; Spassov 1989). The opinion that *Canis lupaster* must be considered as a different taxonomic unit was recently confirmed by other studies based on morphological differences (Spassov and Stoyanov 2014; Bertè 2017). In our previous study we found that skulls from Algeria assigned to *Canis lupaster* are quite different from *Canis aureus* (from Europe and Africa, formerly considered as one species) and *Canis lupus* not only morphometrically but also morphologically (Spassov and Stoyanov 2014), suggesting significant morphological variation and the presence of at least two different forms of *Canis lupaster* in Africa. Morphologically, *Canis lupaster* skulls resemble jackals more than wolves but are bigger and with different proportions (Spassov and Stoyanov 2014; Bertè 2017). Koepfli et al. (2015) made a similar suggestion, specifically with regards to the shape ratio comparison, analyzing the morphological data originally reported by Van Valkenburgh and Wayne (1994). Field observations in Senegal allowed Gaubert et al. (2012) to provide a morphological and behavioural diagnosis of the African wolf that clearly distinguished it from the sympatric golden jackal. However, mitochondrial DNA analyses identified *Canis lupaster* haplotypes in African jackals from Senegal, questioning the genetic differentiation between the proposed African wolves and African golden jackals (Gaubert et al. 2012). Unlike the molecular-based taxonomy which assumes only one species across North Africa, the data of Saleh and Basuony (2014) shows considerable diversity within that genus in Egypt and Libya. The authors suggested the wolf-like canid species known only from the Nile Delta and Nile Valley to be named *Canis lupaster doederleini*. The name *Canis doederleini* Hilzheimer, 1908 was also suggested by Gonzalez (2012) for the population of larger wolf-like canid species from Nile Valley instead of *Canis lupaster*. According to the same author, there are two different taxa of wild *Canis* in this general area, but before allocating scientific names to them it is necessary to return to the original descriptions of *Canis lupaster* and other taxa described from the region, and to the type material. Considering also the valuable attempt of Gaubert et al. (2012) in integrating behavioural and genetic data on Senegal canids, Gippoliti (unpublished) suggests that, as far as alpha taxonomy is concerned, the “*Canis anthus*” complex (the African golden jackals, or African golden wolves as they have been termed), cannot be subsumed into a unique species occurring over the whole vast and highly ecologically diverse territory, but they are represented by multiple lineages (putatively species), perhaps originating from different waves of colonization from Eurasia. He suggests that the hypothesis of two species (*Canis anthus* for the smaller canids and *Canis lupaster* for the larger ones) already proposed (see de Beaux 1923) should be a better starting point for a revision of the group (see also Bertè 2017). A similar conclusion was proposed by Kryštufek and Tvrtković (1990), who referred to skulls assigned to *Canis aureus* as African material other than *Canis aureus lupaster*. In the study of Van Valkenburgh and Wayne (1994) the specimens from different populations of African golden jackal are considered all together but the authors recognise that the population of North Africa is quite different.

African golden jackals (here referred to as *Canis anthus* s. str.) could be easily separated from the Eurasian specimens by their skull shape and length of upper carnassial (P^4^). Differences in skull shape and dental morphology could be explained by their food preferences. Longer carnassial teeth are usually correlated with a more carnivorous diet (Van Valkenburgh and Wayne 1994). The Eurasian golden jackal *Canis aureus* diverged earlier from the *Canis lupus* plus *Canis latrans* clade, about 1.9 mya, than the African golden jackal *Canis anthus*. The divergence between the African lineage of golden jackals and the grey wolf plus coyote clade was estimated at 1.3 mya (Koepfli et al. 2015). African jackals (here referred to as *Canis anthus* s. str.) form a homogenous cluster despite the origin of specimens. There are no differences in shape and size of skull among jackals from Libya, Tunisia, Sudan and Ethiopia. These results raise the question about the existence of more than two subspecies in North Africa as suggested by Moehlman and Hayssen (2018), but it depends on acceptance of the proposed taxonomic status of *Canis lupaster* (Rueness et al. 2011; Gaubert et al. 2012; Koepfli et al. 2015; Gopalakrishnan et al. 2018).

A recent comprehensive study of African and Eurasian golden jackals, based on mitochondrial and nuclear genome sequences, has found strong support to merit the recognition of *Canis anthus* as a genetically distinct canid species that diverged approximately 1.3 million years ago from related grey wolves (Koepfli et al. 2015). The authors also compared morphologically Eurasian and African golden jackals, based on a re-analysis of the morphometric data originally collected by Van Valkenburgh and Wayne (1994), and found that they were similar, but their sample did not include European specimens of *Canis aureus*. A recent genetic study included a larger and more geographically widespread sampling of African golden jackal and also showed that *Canis anthus/lupaster* was distinct from the Eurasian *Canis aureus* (Gopalakrishnan et al. 2018). Based on molecular sequencing and morphological analyses, Viranta et al. (2017) suggested that the estimated current geographic range of golden jackal in Africa represents the African wolf range, but considered *Canis anthus* (Cuvier, 1820) as *nomen dubium* and proposed *Canis lupaster* as the name for the African wolf. However, an exhaustive analysis on different populations of African golden jackal is absent. In terms of conservation, it appears urgent to further characterize the status of the African wolf with regard to the African golden jackal (Gaubert et al. 2012). My results are consistent with recent genetic (Gaubert et al. 2012) and morphometric studies (Kryštufek and Tvrtković 1990; Gonzalez 2012; Spassov and Stoyanov 2014; Saleh and Basuony 2014; Bertè 2017) and suggest that at least two different morphotypes of *Canis lupaster* exist in North Africa. Nonetheless, the question still remains as to whether the larger canid that has been commonly known as the wolf-like jackal (Flower 1932); the Egyptian jackal (Clutton-Brock et al. 1976); and the wolf-jackal (Kurtén 1965), should be considered as a different taxonomic unit following the proposal of Spassov (1989). Some authors identified it as *Canis lupaster* (Hemprich and Ehrenberg 1833; Anderson 1902; Hilzheimer 1908; Flower 1932) or *Canis lupus lupaster* (Ferguson 1981), but it is an open question that requires genetic and morphological analyses of a comprehensive and geographically representative set of samples and specimens. I suggest a taxonomic revision, but extensive research needs to be done on genetics, morphology, biogeography, behaviour and ecology.

## Conclusion

Multivariate analyses revealed that jackal specimens of *Canis aureus* sensu lato, included in this study, formed three very clearly distinct clusters in shape space: European jackals, including Anatolia and the Caucasus, African golden jackals and African wolves. There was no pronounced geographic variation in skull size and shape among the specimens from Europe and Asia Minor. These results support the opinion of Spassov (1989) that jackals from Europe, including those from the Dalmatian coast, Anatolia and the Caucasus belong to one subspecies: *Canis aureus moreotica* (I. Geoffroy Saint-Hilaire, 1835). Although with some overlap, Dalmatian jackals could be very well separated from the other Eurasian and African golden jackals by LDA, giving further support for the continuous presence of ancient populations along the Dalmatian coast (Fabbri et al. 2014; Rutkowski et al. 2015). The present study confirmed morphometrically that all jackals included so far in the taxon *Canis aureus* may represent three taxa of canids and supports the hypothesis that at least two different taxa (species?) of *Canis* occur in North Africa, raising the question about the need for further genetic, morphological, behavioural and ecological research to resolve the taxonomic uncertainty. These results are consistent with recent genetic and morphological studies and give further insights on golden jackal (*Canis aureus*) taxonomy. Understanding the species’ phylogeny and taxonomy is crucial for the conservation and management of the expanding golden jackal population in Europe.

## Supplementary materials

Supplementary material 1 Origin and catalogue number of measured skulls Data type: list of specimens. Explanation note: The list includes information about museum ID, source, catalogue number, collector's name (if available), species identification, origin, sample locality, and sex of all individuals, used in the analyses. https://binary.pensoft.net/file/387817This dataset is made available under the Open Database License (http://opendatacommons.org/licenses/odbl/1.0/). The Open Database License (ODbL) is a license agreement intended to allow users to freely share, modify, and use this Dataset while maintaining this same freedom for others, provided that the original source and author(s) are credited.Stoyan Stoyanov
